# Spine Calcium Transients Induced by Synaptically-Evoked Action Potentials Can Predict Synapse Location and Establish Synaptic Democracy

**DOI:** 10.1371/journal.pcbi.1002545

**Published:** 2012-06-14

**Authors:** David C. Sterratt, Martine R. Groen, Rhiannon M. Meredith, Arjen van Ooyen

**Affiliations:** 1 Institute for Adaptive and Neural Computation, School of Informatics, University of Edinburgh, Edinburgh, Scotland, United Kingdom; 2 Department of Integrative Neurophysiology, Center for Neurogenomics and Cognitive Research, VU University Amsterdam, Amsterdam, The Netherlands; Indiana University, United States of America

## Abstract

CA1 pyramidal neurons receive hundreds of synaptic inputs at different distances from the soma. Distance-dependent synaptic scaling enables distal and proximal synapses to influence the somatic membrane equally, a phenomenon called “synaptic democracy”. How this is established is unclear. The backpropagating action potential (BAP) is hypothesised to provide distance-dependent information to synapses, allowing synaptic strengths to scale accordingly. Experimental measurements show that a BAP evoked by current injection at the soma causes calcium currents in the apical shaft whose amplitudes decay with distance from the soma. However, *in vivo* action potentials are not induced by somatic current injection but by synaptic inputs along the dendrites, which creates a different excitable state of the dendrites. Due to technical limitations, it is not possible to study experimentally whether distance information can also be provided by synaptically-evoked BAPs. Therefore we adapted a realistic morphological and electrophysiological model to measure BAP-induced voltage and calcium signals in spines after Schaffer collateral synapse stimulation. We show that peak calcium concentration is highly correlated with soma-synapse distance under a number of physiologically-realistic suprathreshold stimulation regimes and for a range of dendritic morphologies. Peak calcium levels also predicted the attenuation of the EPSP across the dendritic tree. Furthermore, we show that peak calcium can be used to set up a synaptic democracy in a homeostatic manner, whereby synapses regulate their synaptic strength on the basis of the difference between peak calcium and a uniform target value. We conclude that information derived from synaptically-generated BAPs can indicate synapse location and can subsequently be utilised to implement a synaptic democracy.

## Author Summary

Neurons receive information from other neurons via hundreds of contacts (synapses) spread across their dendritic branches. Input signals from synapses propagate along a dendrite to the cell body (soma), where the neuron decides whether or not to produce an action potential. Signals that travel further decay more. Were all synapses equally strong, a synapse far from the soma would have less influence on the decision than a synapse close by. However, neurons in the hippocampus, which are involved in learning and memory, have synapses far from the soma that are stronger than those close by, so that all synapses have an equal voice (“synaptic democracy”). But how can a synapse “know” how far it is from the soma? Using a computational model of a hippocampal neuron, we show that the action potential, which propagates from the soma back into the dendrites, contains information with which synapses can estimate their somatic distance. Specifically, the calcium concentration at the synapse, which is modulated by the backpropagating action potential, decreases with distance from the soma. We show that when the strength of a synapse is adapted in a self-organising manner based on calcium concentration, synaptic democracy is obtained.

## Introduction

CA1 pyramidal neurons receive numerous synaptic inputs across their extensive dendritic tree, with synapses located up to hundreds of micrometres from the soma [1]. Due to electrotonic filtering, a distal synapse evokes a smaller EPSP at the soma than a proximal synapse of equal synaptic strength and is therefore less effective at generating somatic action potentials [2]. In CA1 pyramidal neurons, synaptic scaling overcomes this inequality with larger synaptic conductances at distal Schaffer collateral synapses than at proximal synapses [3]–[5]. This makes the amplitude of a synaptic response at the soma independent of its dendritic location, a phenomenon known as ‘dendritic democracy’ [6].

It is not clear what cues synapses may use to establish this distance-dependent scaling along the dendrites but internal activity-dependent signalling by the neuron may provide this information. A likely candidate is the backpropagating action potential (BAP), which decreases in amplitude and arrives later as it travels further along the apical shaft [7], [8]. BAPs activate voltage-gated calcium channels, causing transient, local increases in calcium concentrations at dendritic spines [9], [10]. Previous experiments, including ours, have measured BAPs that were induced artificially in neurons via somatic current injection [7]–[10]. However, *in vivo* action potential generation occurs via synaptic stimulation distributed across the dendritic tree, which could evoke a different spatiotemporal pattern of voltage and calcium concentration at spines. Furthermore, AP propagation speed decreases in dendrites with a smaller diameter, such as distal and oblique dendrites [11]. Thus the relationship between BAP features and distance may vary across different branches of the dendritic tree and depend on previous synaptic activity.

The two available stimulation methods in slice experiments, extracellular stimulation and glutamate uncaging, are not yet able to elicit a physiologically-realistic, synaptically-evoked BAP. The first method, extracellular stimulation, stimulates both glutamatergic and GABAergic axons and requires an artificially large tetanus to induce an AP. The second method, synapse stimulation by laser-induced glutamate uncaging near a spine, requires scanning two-photon laser microscopes that currently are only able to uncage glutamate at ca. 10 spines within a 5 ms time window. This is insufficient to elicit an AP at the soma of CA1 pyramidal cells, which requires many simultaneously-activated synapses. In addition, measuring voltage in dendritic branches with voltage sensitive dyes is difficult, due to their limited signal-to-noise ratio and toxicity [12]. Although direct patch clamp recordings have been made at the apical shaft [3], [13], this is not yet possible for the thin oblique dendrites, where the majority of the spines are located [14]. Therefore we took a modelling approach to investigate whether the calcium and voltage signals associated with synaptically-evoked BAPs contain sufficient information to predict synapse location.

We added spines to a well-established CA1 pyramidal neuron model that contains both active and passive properties distributed across a detailed morphology and that has been verified by combined dendritic and somatic recordings [15]. In addition, we used a range of CA1 morphology reconstructions, so as to exclude potential morphology-specific simulation results. We investigated whether features of the voltage and calcium signals, namely their peak, integral and time of onset, could be used as predictors for synaptic location. A good distance predictor should not only contain reliable distance information but should also give consistent results for different types of stimulation. Importantly, the predictor should be a suitable candidate for homeostatic scaling of synaptic strength. This implies that the value of the predictor should respond to changes in synaptic strength, enabling the system to self-organise into a state of synaptic democracy.

Under *in vivo*-like conditions of synaptic stimulation, in non-scaled CA1 pyramidal neurons, we find that the peak value of calcium transients, but not membrane potential, integral values or onset latencies, is strongly correlated with distance and EPSP attenuation. Interestingly, setting one peak calcium target for all spines and homeostatically regulating synaptic strength on the basis of peak calcium resulted in synaptic democracy. Thus, calcium signals in spines induced by synaptically-evoked action potentials contain distance-dependent information across the CA1 dendritic tree that can be used to set up a synaptic democracy.

## Materials and Methods

### Compartmental model

**Figure 1:**
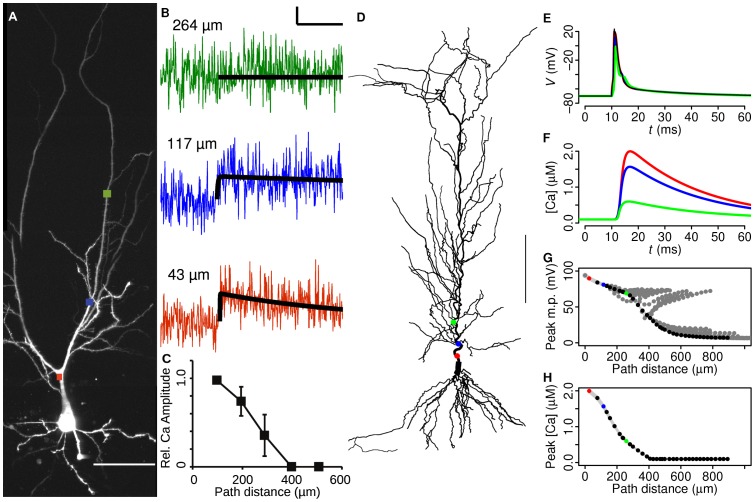
Peak calcium induced by somatic BAP decays with distance in both experiments and model simulation. **A**, Two-photon compressed z-stack morphology of CA1 pyramidal neuron. Squares indicate line-scan measurement sites (scale bar 50 µm). **B**, Corresponding single BAP-induced fluorescence changes in the apical shaft. A double exponential is fitted to the fluorescence traces as described in Materials and Methods. Distances between the point measured and the soma are indicated, determined by tracing the fluorescence of Alexa 594 in 3D from the scanned region back to the soma. **C**, Peak calcium-induced fluorescence plotted against distance to soma. Mean ± S.E.M. shown. Multiple points are measured per cell. Fluorescence amplitude is plotted relative to the first measured data point at circa 100 µm, to show the distance-dependent decrease of amplitude corrected for the large amplitude variation between cells. **D**, CA1 pyramidal neuron morphology of the model (scale bar 100 µm). Circles indicate locations of recordings and points with corresponding colour in E–H similar to the experimental paradigm (A–C). **E**, **F**, Peak voltage and peak calcium levels in the apical shaft following a somatically-initiated BAP. **G**, **H**, Peak voltage and calcium levels versus distance from soma for all apical dendritic shaft locations. Note that distance to soma is measured in 3D along the dendrites, while panel D shows a 3D-compressed image.

A previous morphologically-realistic compartmental model of a hippocampal CA1 pyramidal cell was modified to include Schaffer collateral spines across the dendritic tree [15], [16]. In short, the multi-compartment model includes calcium buffering and the following ionic currents: a voltage-gated sodium current (*I*_Na_), a potassium delayed rectifier current (*I*_KDR_), a fast inactivating, A type potassium current (*I*_A_), a hyperpolarisation-activated mixed cation current (*I*_h_), a LVA T-type calcium current (*I*_CaT_), a HVA R-type calcium current (*I*_CaR_), a HVA L-type calcium current (*I*_CaL_), a calcium-dependent potassium current (*I*_AHP_) and a slowly inactivating potassium current (*I*_m_) [15]. To make the model consistent with our calcium imaging data (Fig. 1B, C), we had to set the density of L-type calcium currents in the proximal apical shaft (first 50 µm from soma) equal to the density in the distal dendrites; this had little effect on the backpropagation of APs in the model. In addition, we applied the model to two other CA1 pyramidal cell morphologies, based on a Neurolucida reconstruction of biocytin-filled neurons. Since no full axon reconstruction was available for these morphologies, we used the axon reconstruction described in the original morphology. The model is implemented in NEURON [17] and the code for all simulations in this paper is available from the ModelDB database (accession number 144490 http://senselab.med.yale.edu/senselab/modeldb).

### Spine distribution and activation

To model synaptic input, spines were distributed at random over the dendrites of the stratum radiatum dendritic section, based on distribution patterns for adult CA1 pyramidal neurons [18]. For each stimulation condition, the simulation was repeated 100 times with a new distribution of synapses. Peak, integral and delay-to-peak (defined as the time from synapse stimulation until the peak signal) were measured for voltage and calcium signals in spines. To ensure sufficient data points per stimulation to determine a reliable mean per synapse, only synapses that were activated 10 or more times were analysed.

Spines were simulated using separate compartments for the neck (diameter 0.2 µm, length 1.0 µm) and spine head (diameter 0.4 µm, length 0.2 µm) [10]. Synaptic NMDA and AMPA receptors and R-type calcium channels were located on the spine head (see below for detailed description). Apart from these receptors and channels, the spines had only passive conductances, the membrane resistance being 10 kΩcm^2^ and the intracellular resistance 50 Ωcm (the same as in the oblique dendrites and proximal apical shaft; see [15]).

Calcium entered into the spine and dendrites through activation of synaptic glutamate receptors and voltage-gated calcium channels. Based on experimental evidence [10] and modelling results [19], we assumed that there was no diffusion of calcium through the spine neck. Accumulation, buffering and extrusion of calcium in the spine head were modelled using first order kinetics [20]:
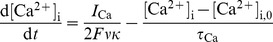
(1)where [Ca^2+^]_i_ is the intracellular calcium concentration, [Ca^2+^]_i,0_ = 70 nM is the resting concentration of calcium, *I*_Ca_ is the total calcium current through the NMDA, AMPA and R-type channels, *τ*_Ca_ = 12 ms is the calcium pump extrusion time constant, *κ* = 20 is the buffer capacity [10], *v* is the volume of the spine head and *F* is Faraday's constant.

### NMDA channels

The time course of NMDA currents were modelled as a sum of exponentials, 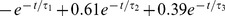, with rise time constant *τ*_1_ = 1.7 ms and decay time constants *τ*_2_ = 68 ms and *τ*_3_ = 444 ms. The time constants derive from excised patch recordings at 22°C [21], corrected for the simulation temperature of 34°C using a *Q*_10_ of 3 [22]. The peak NMDA conductance was *g*_NMDA_ = 45 pS, based on a peak conductance of 70 pS measured in spines in CA1 cells [23] and allowing for a 40% reduction due to the steady-state calcium-dependent NMDA receptor inactivation [24].

Voltage-dependent block was modelled as an instantaneous process, with the fraction of unblocked channels being given by Vargas-Caballero and Robinson [25]:
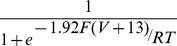
(2)where *R* is the molar gas constant and *T* is the temperature in Kelvin.

The NMDA receptor passed both a nonspecific ion current *I*_M,NMDA_ and a calcium current *I*_Ca,NMDA_ given by:
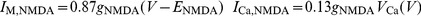
(3)where *E*_NMDA_ = 0 mV is the NMDA reversal potential and *V*_Ca_ is the effective calcium driving force given by the Goldman-Hodgkin-Katz current equation,
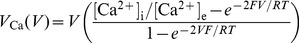
(4)with [Ca^2+^]_e_ being the extracellular calcium concentration of 2 mM. The ratio of *I*_M,NMDA_ to *I*_Ca,NMDA_ derives from the ratio of calcium to caesium permeability in hippocampal CA1 and CA3 [23].

### AMPA channels

The AMPA conductance was modelled by a dual exponential with a rise time constant of 0.2 ms and a decay time constant of 5 ms [26]. The maximum AMPA conductance, g_AMPA_, was 200 pS for all synapses in the non-scaled simulations. This value was based on experimentally measured EPSCs at the apical shaft and the AMPA channel reversal potential [3]. Nonspecific ion and calcium flow through the AMPA channels was modelled in the ratio 99.8%∶0.2% [23].

### R-type calcium channels

The current through the R-type calcium channel was given by:
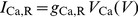
(5)where *g*_Ca,R_ approaches the slope conductance of the channel for large negative voltages.

The kinetics of the R-type channel were taken from the recordings at 22°C [27] and scaled to 34°C using a *Q*_10_ of 3 typical of ion channels [28]. The conductance was
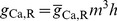
(6)where *m* and *h* are Hodgkin-Huxley state variables obeying first order kinetics. Their steady-state values were
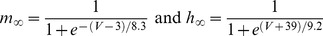
(7)with *V* in mV. The time constants (in ms) of the state variables were
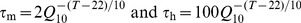
(8)where *T* is temperature in degrees Celsius.

Based on a unitary conductance of 17 pS [27] and 10 channels per spine [29], we took 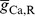 to be 170 pS. With these parameter values, the peak calcium concentration in a spine in response to a somatically-induced BAP was around 1 µM, which is well within the range measured experimentally [10].

### Stimulation protocols

Synaptic inputs were modelled at subthreshold (190 synapses activated) and suprathreshold (240 synapses activated) levels as bursts of synchronous Schaffer collateral activity. To simulate spike jitter that occurs during sharp-wave or theta rhythms, synapses were activated in an asynchronous pattern, randomly drawn from a 10 ms time window [30]. During a single synaptic stimulation episode, inputs were activated only once.

### Attenuation

The attenuation at a synapse is defined as 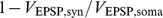, where 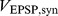 is the amplitude of the EPSP at the synapse and 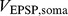 the corresponding amplitude measured at the soma.

### Attenuation-dependent scaling of synapses

The synapses were scaled by multiplying each synapse's conductance in the unscaled simulations by its EPSP attenuation and then dividing by the mean EPSP attenuation of all synapses.

### Homeostatic regulation of synaptic strength

In developing neurons, postsynaptic calcium regulates AMPA trafficking and expression in spines [31], [32]. To investigate whether this can be used to set up a synaptic democracy, we carried out simulations in which the AMPA conductances of activated synapses were adjusted based on the peak calcium levels in the spines. At the start of the simulation, all synapses had the same AMPA conductance (*g*_AMPA_ = 200 pS). For each simulation run, a different set of 240 synapses was activated to induce a BAP. In each run, the AMPA conductance of an activated synapse was updated according to
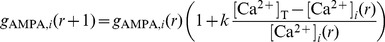
(9)where *g*_AMPA,*i*_(*r*) and [Ca^2+^]*_i_*(*r*) are the AMPA conductance and peak calcium at synapse *i* at run *r*; [Ca^2+^]_T_ is the target peak calcium value, which we set at 47.0 µM, the median of the peak calcium in the scaled synapses simulation described above; and *k* = 0.1 determines the speed with which the AMPA conductance changes. Thus, in each run, the AMPA conductance changes depending on the difference between the target peak calcium and the current peak calcium; 500 runs were sufficient to create stable synaptic strength in all spines.

### Statistical analysis

To quantify the predictive power of each feature *x* for synapse distance or attenuation *y*, we determined the least squares fit of the distance or attenuation to a straight line 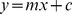 or an exponential 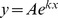. In each case the significant fit which gave the higher *R* ^2^ value was accepted.

### Ethics statement

All animal use was approved by the Animal Welfare Committee of the VU University Amsterdam.

### Two-photon calcium imaging

Young adult male Wistar rats (P28–P42) were decapitated and brain removed in ice cold slice solution containing (in mM):110 choline chloride, 11.6 Na-ascorbate, 3.10 Na-pyruvate, 2.50 KCL, 1.25 NaH_2_PO_4_, 7 MgCl_2_, 0.50 CaCl_2_, 10 glucose, 26 NaHCO_3_ [33]. 300 µm horizontal hippocampal slices were cut using a LEICA VT1000S vibratome. Slices were transferred to a holding chamber containing artificial cerebrospinal fluid (aCSF) containing (in mM): 125NaCl, 3 KCl, 1.2 NaH_2_PO_4_, 10 glucose and 26 NaHCO_3_, and heated at 34°C for 20 minutes before storing at room temperature until recording started. All recordings were made in 32°C aCSF.

Whole cell patch-clamp recordings were made from CA1 pyramidal cells using 2.5–4.5 MΩ glass pipettes filled with intracellular solution containing (in mM): 154 K-gluconate, 1 KCl, 10 HEPES, 4 Mg-ATP, 4 K_2_ phosphocreatine, 0.4 GTP. In some experiments, 0.2% biocytin was added for morphological verification and K-gluconate was adjusted to 148 mM. Pipettes were filled with intracellular solution containing Alexa-594 (80 µM) and the calcium dye, fluo-4 (200 µM) (Molecular Probes, Invitrogen). Series resistance was not allowed to exceed 20 MΩ and was monitored throughout the recording. Fluorescent dyes were allowed to diffuse into the cell for 20 minutes before measurements began.

Dendrites were line-scanned bidirectionally at a frequency of 8 kHz, at various distances from the soma, using a LEICA RS2 two-photon laser scanning microscope with a 63× objective and a Ti∶Sapphire laser tuned to 830 nm excitation. Action potentials were elicited in the soma by a 50 ms current pulse. Relative fluorescence changes are given as the percentage change of Fluo-4 fluorescence from baseline relative to the stable, voltage-independent Alexa-594 fluorescence as described before [34]. Three traces were averaged per distance. A double exponential 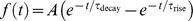 was fitted to the signal to determine the rise, decay and peak amplitude of the fluorescence signal. Fits were regarded as significant when *A* was significant with a 99% confidence interval.

At the end of the experiment, a z-stack was made of the neuron, to reconstruct the dendritic tree. Using the open source program ImageJ [35], the region of the dendritic tree scanned was traced back to the soma in 3D based on fluorescence to determine the actual distance travelled by the BAP.

In some experiments, the recorded cell filled with biocytin was fixed in 4% paraformaldehyde at the end of the experiment, and processed immunohistochemically with chromogen 3,3′diaminobenzidine tetrahydrochloride using the avidin–biotin–peroxidase method. Two different CA1 pyramidal neuron dendritic morphologies were selected for manual reconstruction using Neurolucida (MicroBrightField). Neurolucida reconstructions were directly imported into NEURON and are publicly available together with the code of the model.

## Results

### Action potential back propagation following somatic current injection

We measured BAP-induced calcium currents experimentally, using multi-photon calcium imaging in hippocampal horizontal slices (P28–P42). Calcium concentrations were measured in the dendritic apical shaft at different distances after a BAP in CA1 hippocampal cells was induced by somatic current injection (Fig. 1A). The BAP-induced amplitude of the calcium signal decreased with distance along the apical shaft up to 400 µm, in accordance with previous reports in mature CA1 hippocampal neurons (Fig. 1B, C, [7], [8]). This suggests that information about distance, required to set up a dendritic democracy, could be provided by calcium concentration.

However *in vivo*, action potentials are evoked by synaptic inputs rather than by current injected directly into the soma. Synaptic activity in the dendrites, which alters local dendritic excitability, affects propagation of the resultant BAP into the same dendritic region. Because slice experiments do not allow investigation of synaptically-evoked BAPs, we adapted a realistic morphological and electrophysiological model of a CA1 pyramidal cell (Fig. 1D) [15], [16]. Stimulating the model at the soma by current injection, mimicking the experimental data described above, showed a similar inverse relationship between local calcium influx at the apical shaft and dendritic distance to that found in the experiment (Fig. 1H). We therefore proceeded to use the model to test the effect of synaptically-induced action potentials.

### Peak calcium concentration is the best correlate of synaptic distance from the soma for synaptically-driven backpropagation

**Figure 2:**
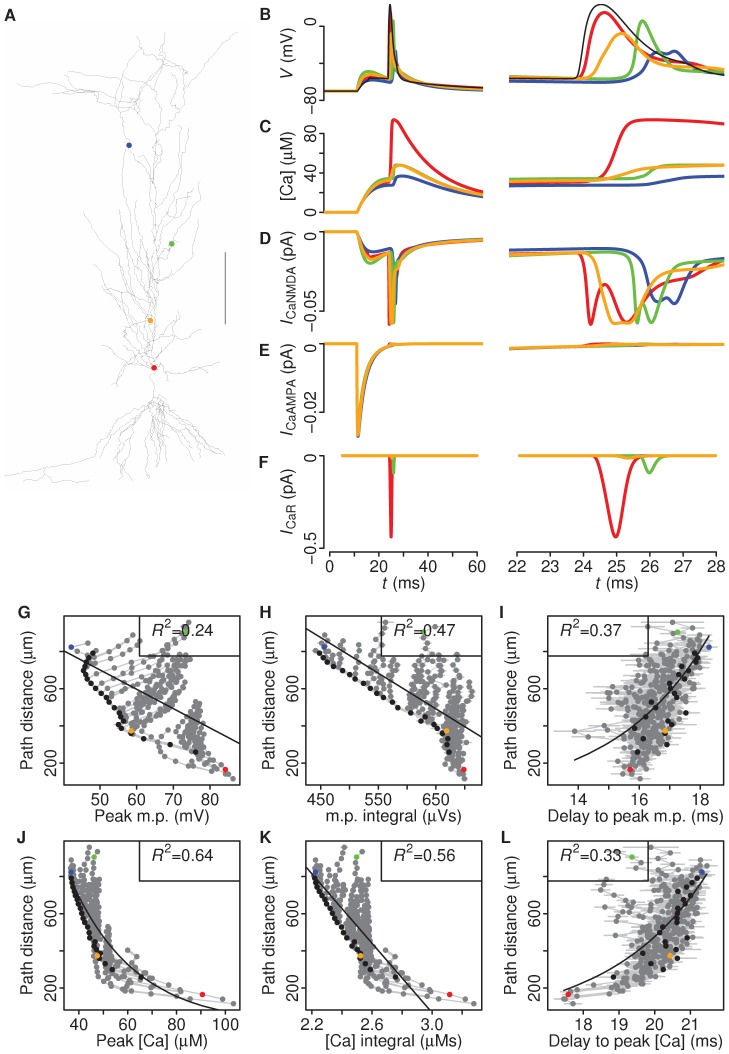
Synaptic induction of BAP displays distance-dependent voltage and calcium changes across the dendritic tree. **A**, CA1 model neuron. Circles indicate spine head locations of example measurements highlighted in B–L. **B–F**, Measurements from the spine heads indicated by the corresponding colours in panel A, indicating membrane potential (**B**), calcium concentration (**C**), NMDA-mediated currents (**D**), AMPA-mediated currents (**E**) and R-type currents (**F**). The right-hand plots show the same data, but magnified around the time of the arrival of the BAP at the spines. **G–L**, Peak, integral and delay-to-peak voltage and calcium measurements are plotted as predictors for path distance. Black circles indicate apical shaft spines. *R* ^2^ values are shown.

To test what effect synaptic stimulation could have on backpropagation, BAPs were generated via synchronous synaptic activation across the model CA1 neuron's dendrites (Fig. 2A). Voltage and calcium concentration were measured in the spines. Synaptically-induced BAPs generated a markedly different pattern of spatial-temporal kinetics than somatically-induced BAPs (Fig. S1), with a smaller and lower range of peak calcium values for somatic induction alone compared with synaptic stimulation (Fig. S1M).

With synaptically-induced BAPs, synaptic stimulation caused an initial depolarisation and influx of calcium into activated spines via AMPA receptors and partially-unblocked NMDA receptors (Fig. 2B–F). Arrival of the BAP at the spine head produced an additional increase in local membrane potential and calcium concentration via further unblocking of NMDA receptors and opening of R-type voltage-gated calcium channels (Fig. 2B–F). Because R-type channels have a high threshold for activation, the calcium influx through R-type channels is more prominent in proximal synapses than in distal synapses, in which the calcium influx is mediated mainly through NMDA receptors (Fig. 2F).

We next tested whether features of the voltage or calcium signals could provide a distance measure to the spines under these *in vivo*-like stimulation conditions. We looked at the peak value, the integral and the delay-to-peak of the calcium and voltage signals. Surprisingly, peak voltage induced by the synaptically-driven BAP was not a good correlate of synaptic distance, accounting for only 24% of the variance (Fig. 2G). Although the peak voltage decreased with distance along the apical shaft, in agreement with experimental observations [13], [36] and model predictions for passive dendrites [11], [37], there was no good overall correlation of peak voltage with distance. This can be explained by a difference in BAP spread into the apical and oblique dendrites. In the apical dendritic spines, peak voltage decreased with path distance from the soma but this relationship was reversed when the BAP entered the oblique dendritic spines (Fig. 2G). Consequently, for a given BAP amplitude, there was a considerable range of potential synapse distances, e.g. an amplitude of 70 mV only localised the path distance between synapse and soma to within a range of 200–850 µm.

In contrast, peak calcium levels in the stimulated spines were good correlates of soma-synapse distance, with an exponential fit explaining 64% of the variance (Fig. 2J). Unlike peak voltage, peak calcium concentrations in oblique dendritic spines did not increase strongly relative to those in spines on the apical shaft. Besides the peak values, we also looked at the integrals of the voltage and calcium signals in the spines. The integral of membrane voltage showed a weaker correlation with distance (*R* ^2^ = 0.47, Fig. 2H) than peak calcium but substantially higher than peak membrane voltage. Distal synapses showed lower integral values than proximal synapses. Intriguingly, the integral of membrane potential was relatively constant within an oblique dendrite, while synapses along the apical shaft showed strong distance-dependent modulation of the membrane potential integral. The calcium integral showed a similar distance-dependent pattern, although with a higher correlation (*R* ^2^ = 0.56, Fig. 2K).

We further tested whether the time delay from onset of synaptic stimulation to BAP arrival at the spine was a good correlate for soma-synapse distance. Both delay-to-peak of BAP voltage and delay-to-peak of calcium were only weakly correlated with synaptic location (*R* ^2^ = 0.37 and 0.33, Fig. 2I, L, respectively). Thus, for synaptically-stimulated BAPs, peak calcium at the spine is the best correlate of distance from the soma.

### Simulation results are robust to different dendritic morphologies and AMPA conductances

**Figure 3:**
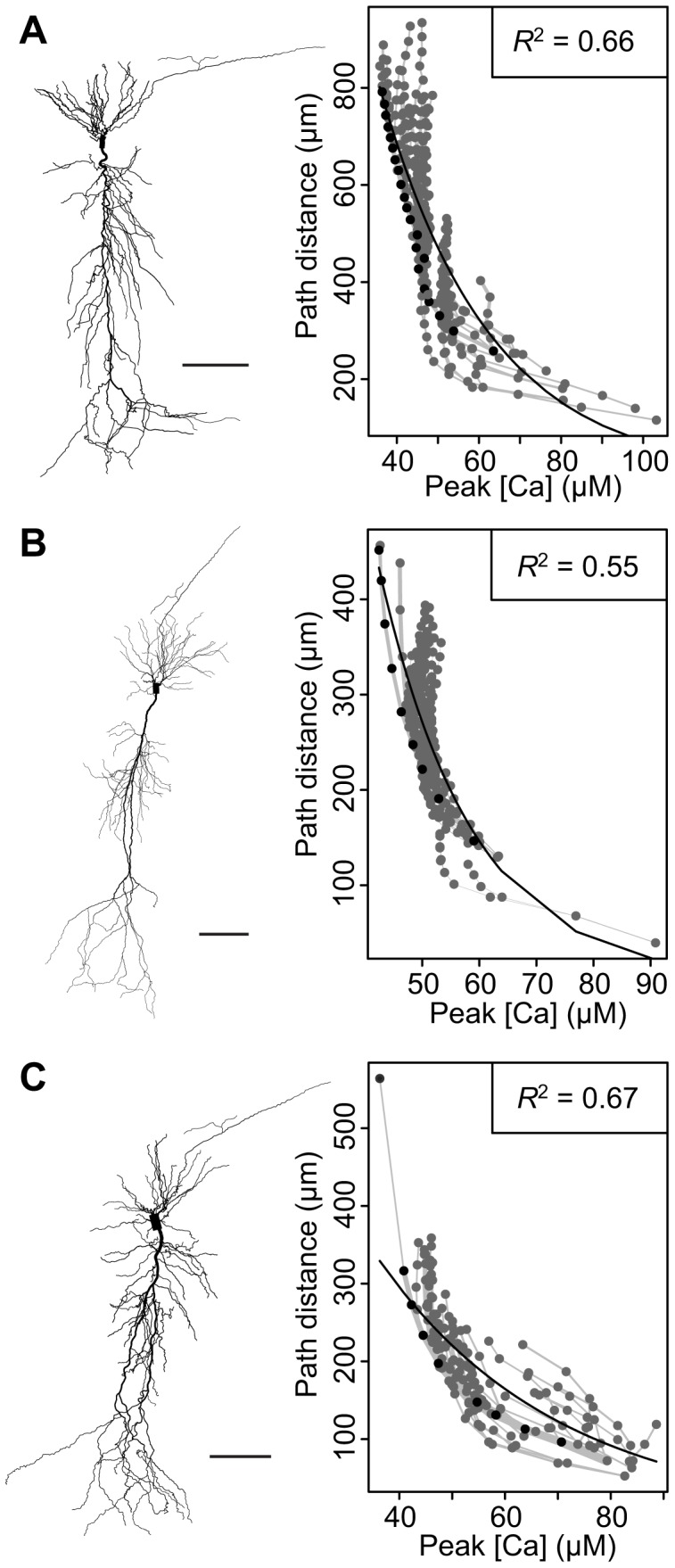
The model is robust to morphological variations. Three different CA1 morphology reconstructions used to test robustness of our simulations. **A**, *left panel*. CA1 morphology redrawn from data provided in Poirazi et al. (2003) and used in the other figures of this paper. **B**, **C**, *left panels*. Two different CA1 neurons filled with biocytin, processed and reconstructed in Neurolucida. Neurons came from hippocampal slice preparations of P28–42 rats. The axon is the same for all cells, taken from Poirazi et al. (2003). Scale bars 100 µm. **A–C**, *right panels*. Peak calcium concentration correlated with dendritic distance from soma for each neuronal reconstruction, with corresponding *R* ^2^ values given.

CA1 pyramidal neurons exhibit a wide variety of morphologies, all characterised by a thick apical shaft with small oblique dendrites at the sides. To test whether morphological variation in CA1 pyramidal cells would influence our simulation results, we repeated the simulation with the two other morphologies described in Fig. 3. Across the different morphologies, peak calcium was consistently moderately or strongly correlated with distance for all CA1 morphologies (*R* ^2^ = 0.55 and 0.67 compared to *R* ^2^ = 0.66 of the original morphology) (Fig. 3). Therefore, morphological variation did not affect our main finding: namely, peak calcium levels in the spines after a synaptically-induced BAP can consistently provide distance information to the synapse. We also tested our model with a two-fold increase of the AMPA conductance (*g*_AMPA_ = 400 pS instead of 200 pS), which yielded similar results (Fig. S2).

### Effect of asynchronous and subthreshold stimulation upon BAP-evoked features

Hippocampal pyramidal neurons exhibit highly synchronised firing patterns during cholinergic activation, with synaptic inputs phase-locked to the local network oscillation [38]. Within this phase-locked period, there is asynchronous jitter in the onset of synaptic currents [30], [39]. In between these bursts, cells are silent and receive only subthreshold activation [40] (i.e. activation that does not trigger an action potential at the soma and consequently no BAP). Therefore, we tested whether the calcium and voltage features correlated with dendritic distance when synapses were activated either asynchronously or under subthreshold conditions.

**Figure 4:**
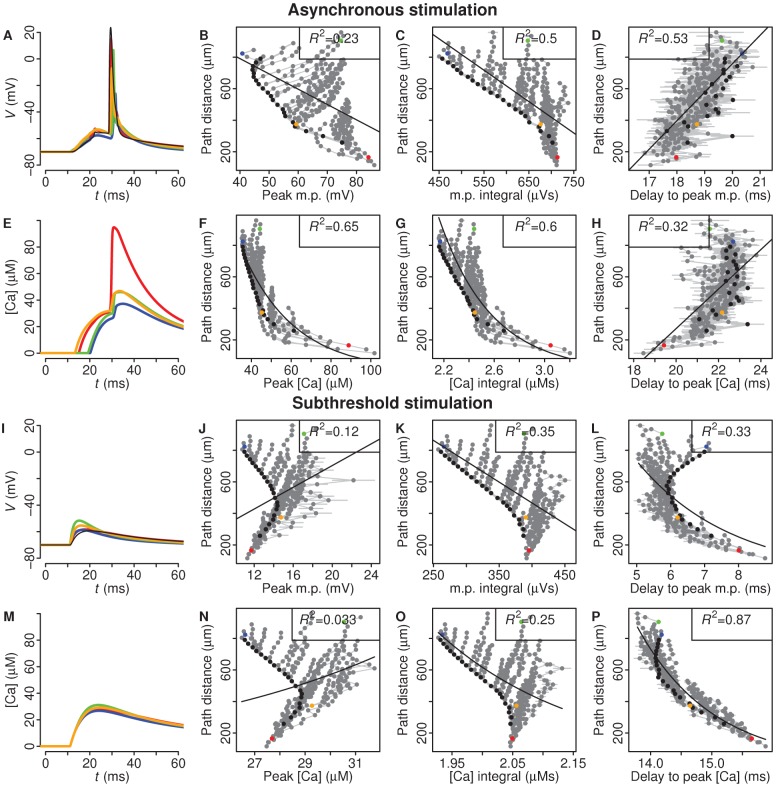
The effect of asynchronous inputs and subthreshold inputs on features tested. Colours indicate spine locations shown in Fig. 2. **A–H**, Asynchronous inputs (240 synapses): In each of 100 simulations, the cell was presented with synaptic inputs whose activation times were randomly drawn from a 10 ms window. **A**, **E**, Example voltage and calcium traces of spines indicated in Fig. 2A. **B–D**, **F–H**, Peak, integral, and delay-to-peak voltage and calcium changes at all asynchronously-activated spines across the dendritic tree plotted against path distance. **I–P**, Subthreshold inputs (170 synapses): **I**, **M**, Synaptically-stimulated changes in voltage and calcium at selected spines (see Fig. 2); **J–L**, **N–P**, Peak, integral and delay-to-peak voltage and calcium changes in spines following subthreshold stimulation plotted against path distance.

As with synchronous synaptic stimulation, peak voltage in asynchronously-activated spines was weakly correlated with distance (*R* ^2^ = 0.23, Fig. 4B). Both delay-to-peak voltage and delay-to-peak calcium also showed moderate or weak correlation with distance (Fig. 4D, *R* ^2^ = 0.53, Fig. 4H, *R* ^2^ = 0.32). Integral voltage and integral calcium continued to contain a moderate amount of distance information (Fig. 4C, *R* ^2^ = 0.50, Fig. 4G, *R* ^2^ = 0.60). However, these correlations were still lower than peak calcium, which remained the strongest correlate with dendritic distance (Fig. 4F, *R* ^2^ = 0.65).

To investigate subthreshold activation, the number of activated synapses was reduced so that a BAP was no longer generated at the soma. Peak voltage continued to convey no accurate distance information (Fig. 4J, *R* ^2^ = 0.12). Peak calcium was not informative anymore and the range of calcium concentration dropped to 26–32 µM compared to 30–90 µM in the BAP case (Fig. 4N, *R* ^2^ = 0.03 and 2J). Voltage and calcium integrals maintained their spatiotemporal pattern, although with lower correlations (Fig. 4K, *R* ^2^ = 0.35, Fig. 4O, *R* ^2^ = 0.25, compared with Fig. 2H, K).

The delay between the onset of the stimulus and reaching peak calcium concentration was highly correlated with distance, albeit in the opposite direction from that in suprathreshold activation (*R* ^2^ = 0.87, Figs. 4P and 2L). The delay-to-peak voltage showed no distance discrimination after 400 µm (Fig. 4L). Thus, at subthreshold stimulation, delay-to-peak calcium but not actual peak calcium was the best correlate of distance.

### Mechanism underlying the correlation of peak calcium with synaptic distance

**Figure 5:**
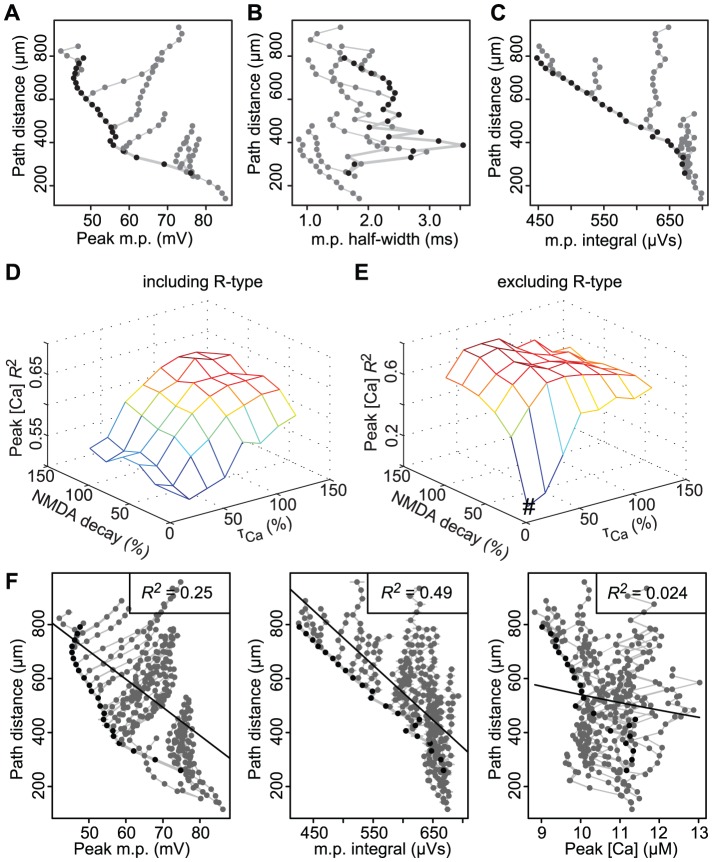
The contribution of calcium conductance and dynamics to distance-dependent correlations. **A**, Relationship between peak voltage and soma-spine distance in a sample of branches in the CA1 model neuron. **B**, Relationship between voltage half-width and distance to the soma for same sample of branches shown in A. **C**, Relationship between voltage integral and distance to the soma for same sample of branches shown in A. **D**, **E**, *R* ^2^ values for combinations of NMDA decay and calcium extrusion time constants for all spines, with and without R-type channels. # indicates an 80% decrease in NMDA decay and in calcium extrusion time constants that are used in all other figures in the manuscript. **F**, For 80% decrease in values (indicated # in E), relationship between soma-spine distance with peak voltage (*left panel*), voltage integral (*centre panel*) and peak calcium in spines (*right panel*). Correlations for peak voltage and voltage integral remain stable (compared with Fig. 2G, H) but the correlation with peak calcium concentration disappears (comparison with Fig. 2J).

Why is BAP-induced peak calcium consistently the best correlate of soma-synapse distance? Following synapse activation, the initial calcium influx into spines is mediated by AMPA receptors and NMDA receptors that have been partially unblocked by the synaptically-induced depolarisation (Fig. 2B–F). Arrival of the BAP at the spine head causes an additional increase in local membrane potential and calcium influx, via further unblocking of NMDA receptors (Fig. 2B–F). NMDA receptors act as coincidence detectors [41] and require, besides synaptic stimulation, postsynaptic depolarisation, in this case provided by the BAP, to unblock the channel further. This calcium increase due to the BAP is responsible for the distance dependency of peak calcium. Since the calcium influx is dependent on membrane potential (see Eqns. 3–7, Materials and Methods), and the BAP amplitude decreases with distance in the apical shaft (Fig. 2G, Fig. 5A), in agreement with experimental findings [13], [36], there is less BAP-induced calcium influx at distal synapses in the apical shaft than at proximal synapses (Fig. 2C, J). Without a BAP, as in subthreshold stimulation, the NMDA channels remained closed and peak calcium is no longer informative about distance (Fig. 4N).

Although the amplitude of the BAP decreases with distance in the apical shaft, peak voltage is not an accurate correlate of synaptic distance for all synapses (Fig. 2G). This is due to the BAP amplitude increasing again when the BAP enters the thin oblique dendrites (Fig. 2G, Fig. 5A). Because of the tapering and sealed end of the obliques, peak voltage increases towards the distal end of the oblique dendrites [42] (Fig. 2G, Fig. 5A), similar to effects seen in sealed ends of axons [43]. Simultaneously with the increase in peak voltage, however, the width of the voltage signal decreased in the oblique dendrites (Fig. 5B), so that the integral of voltage remained relatively constant (Fig. 2H, Fig. 5C). This narrow voltage signal in the thin oblique dendrites shown by our simulations is in agreement with findings from voltage-sensitive dye studies in thin basal dendrites [44]. The different behaviour of the integral of voltage in the obliques, as compared with peak voltage, is why the integral of voltage gives a stronger overall correlation with distance (Fig. 2H, Fig. 5C).

Biologically, the integral of voltage is read out by calcium [41], [45]. Due to the slow time constants involved in calcium extrusion (see Eqn. 1) and calcium influx through NMDA channels (see Eqns. 2–4), the calcium concentration effectively reflects the integral of voltage. Peak calcium therefore also correlated well with synaptic distance (Fig. 2J). A combination of lower time constants for the NMDA channel and a quickening of the calcium extrusion with and without the elimination of R-type channels abolished the distance-dependent correlation with peak calcium (Fig. 5D, E, F, *right panel*) whilst the correlations with peak voltage and voltage integral were not affected (Fig. 5F, *left and centre panels*).

Although the R-type voltage-gated calcium channels, which have fast kinetics, contribute to the calcium influx, especially in the proximal synapses, the influx through the NMDA receptors alone is sufficient to create the distant-dependency of peak calcium, as can be seen when the R-type channels are removed (Fig. 5E, Fig. S3).

### Peak calcium and integrals of voltage and calcium correlate with EPSP attenuation

**Figure 6:**
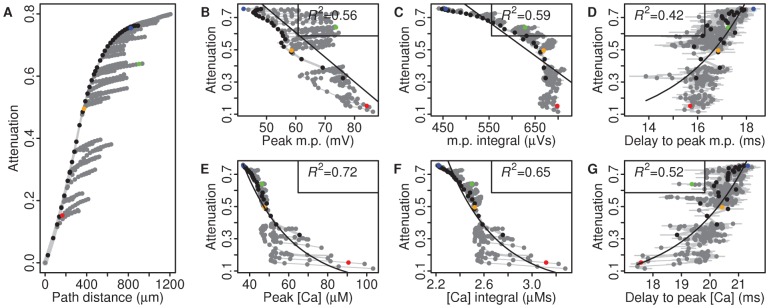
Peak spine calcium following backpropagation strongly correlates with EPSP attenuation. **A**, Relationship between path distance to soma and EPSP attenuation. Coloured circles indicate selected spines (see Fig. 2). **B–G**, Peak, integral and delay-to-peak voltage and calcium signals as correlates for EPSP attenuation. *R* ^2^ values indicated for each fit.

From a functional perspective, a more important measure than path distance of a synapse from the soma is the amount of attenuation EPSPs undergo en route to the soma. Although a major factor influencing EPSP attenuation is distance, there are also other factors involved such as dendritic diameter, the activation state of the dendrite and low threshold voltage-gated channels. To test whether attenuation can also be predicted by BAP features, we measured EPSP attenuation for each synapse, defined as the difference in EPSP amplitudes at the synapse and soma, divided by the EPSP amplitude at the soma (see Materials and Methods). As expected it increased with distance from the soma, though the rate of increase with distance reduced in the smaller oblique branches (Fig. 6A). Attenuation depended on the BAP features in the same way as soma-dendritic distance. Again, peak calcium had a strong predictive power (*R* ^2^ = 0.72, Fig. 6E) while peak voltage and delay-to-peak voltage and calcium were moderately to strongly-correlated (Fig. 6B, D, G). This demonstrates that BAP-induced calcium levels can predict not only soma-synapse distance but also the more physiologically-relevant EPSP attenuation. The integrals of calcium and voltage were also strongly correlated with EPSP attenuation (Fig. 6C, *R* ^2^ = 0.59, Fig. 6F, *R* ^2^ = 0.65) due to low variation in attenuation along each oblique dendrite (Fig. 6A).

### Peak calcium concentration in spines can establish a synaptic democracy

Peak calcium is correlated with distance and could potentially be used by the synapse as distance indicator in setting up a synaptic democracy. For a democracy to be established in a self-organising manner, the feature upon which synaptic strengths are scaled should in itself be responsive to changes in synaptic strength, otherwise synaptic strengths would not stabilise. Therefore, we investigated whether peak calcium, which correlated with distance in non-scaled synapses, is influenced by synaptic strength.

**Figure 7:**
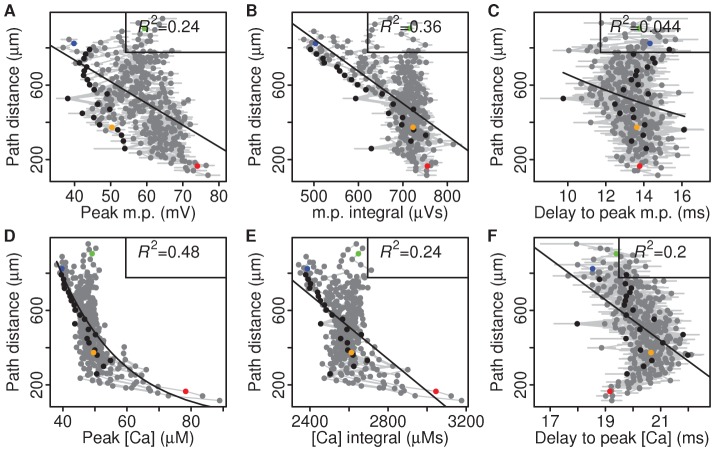
Peak calcium is less correlated with distance once synapses are scaled according to synaptic democracy. **A–F**, Peak, integral and delay-to-peak voltage and calcium signals correlated with for path distance. Coloured circles indicate selected spines (see Fig. 2). *R* ^2^ values indicated for each fit.

To test this, we scaled the strength of synapses, defined as the size of the synapse's AMPA conductance, according to EPSP attenuation (Fig. 6), to mimic synaptic democracy and then investigated the resulting distance-dependency of peak calcium (Fig. 7). While peak calcium showed a clear distance-dependency in the non-scaled simulations, this became less prominent in the scaled scenario (Fig. 7D, Fig. 2J, *R* ^2^ = 0.48 compared to *R* ^2^ = 0.64), indicating that peak calcium was influenced by synaptic strength and reflected in significantly different distributions of peak calcium (Kolmogorov-Smirnov test, *p*<0.001). The other features showed similar or lower correlations with distance as the non-scaled simulations (Fig. 7A–C, E, F, Fig. 2). Thus, peak calcium is influenced by synaptic strength and could therefore potentially be used to regulate synaptic strength.

Interestingly, two recent experimental studies showed that peak calcium can regulate the amount of AMPA receptors in a homeostatic manner. Decreased levels of postsynaptic calcium resulted in an increased amount of AMPA receptors via retinoic acid [32]. Conversely, increased levels of activity at individual synapses resulted in a decreased amount of AMPA receptors, in an NMDA-dependent manner [31].

**Figure 8:**
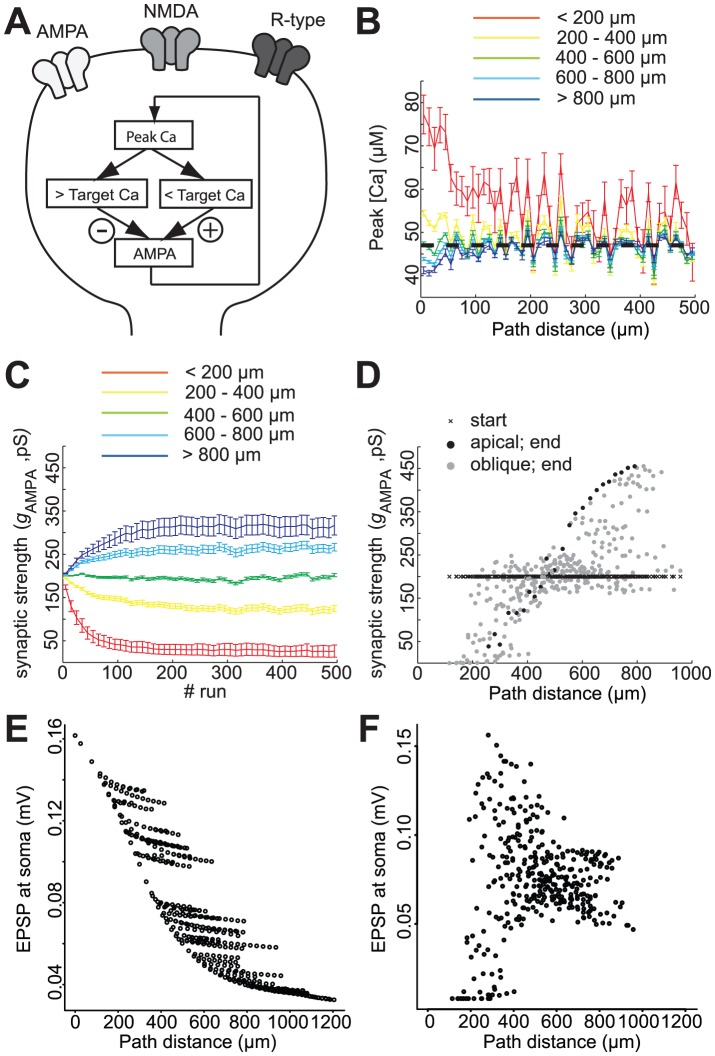
Synaptic democracy established using homeostatic rules based on peak calcium levels. **A**, Schematic showing the homeostatic regulation of synaptic strength, defined as AMPA conductance, by peak calcium. **B**, Peak calcium in spines during the time-lapse homeostatic simulation. Synapses are colour-coded and grouped according to distance. The black dotted line indicates the target level. **C**, Synaptic strength stabilises during a simulation run of 500 trials. As in B, synapses are grouped and colour-coded according to distance. **D**, Synaptic strength at the beginning (black crosses) and at the end of the scaling simulation (closed circles) plotted against distance. Black circles indicate the apical shaft, grey circles the oblique dendrites. **E**, EPSP amplitude at the soma of individual synapses plotted against distance at the start of the simulation. **F**, EPSP amplitude at the soma of individual synapses plotted against distance at the end of the simulation.

We investigated whether homeostatic scaling governed by peak calcium levels could produce a synaptic democracy. Based on the results of the scaled synapse simulations described above (Fig. 7D), we set the target peak calcium value (see Eqn. 9) for all spines at 47.0 µM. Initially, all synapses had the same strength (i.e., AMPA conductance). After each simulation run, the synaptic strength of each synapse was increased or decreased depending on whether the peak calcium value in the spine was lower or higher, respectively, than the target peak calcium level (Fig. 8A, Eqn. 9). Note that in this simulation, synapses were updated once they were activated regardless of whether the response was sub- or suprathreshold (Fig. S4). After 500 runs, the synaptic strengths had reached a stable arrangement in which the distal synapses had higher synaptic strengths than the proximal ones (Fig. 8 C, D, Fig. S4).

Why was this pattern of synaptic strengths produced? When peak calcium in a spine is below the target value, the AMPA conductance of that synapse is increased (Fig. 8A). The next time the synapse is activated and a BAP arrives, the higher AMPA conductance leads to a higher postsynaptic depolarisation and consequently a higher calcium influx via the NMDA channels. During subsequent synaptic activation, the AMPA conductance is further increased until the peak calcium is on average at the target value. The opposite changes occur when the peak calcium starts below the target value. Since the target peak calcium is the same for all synapses, and peak calcium decreases with distance from the soma (with unscaled synapses), distal synapses end up with higher AMPA conductances than proximal synapses.

To test whether the homeostatic scaling had produced a true synaptic democracy, we looked at the EPSP size at the soma. With all synapses having the same strength, EPSP amplitude at the soma depended on the distance of the synapse (Fig. 8E). With the homeostatically-scaled synapses, the EPSP size measured at the soma was mostly distance-independent (Fig. 8F), indicating the establishment of a synaptic democracy. A minority of synapses, namely the most proximal synapses, were not scaled. The density of R-type calcium channels is relatively high for proximal synapses, so that the calcium influx through these channels already resulted in calcium concentrations above the target value. For these spines, the AMPA conductance continued to decrease, as the peak calcium continued to remain above the target value. These spines (2.9% of total spines) converged towards zero synaptic strength and as a consequence, gave rise to very low EPSP values at the soma (Fig. 8 C, D, F). However, for the majority of CA1 pyramidal synapses, homeostatic scaling based on peak calcium concentrations is able to produce a synaptic democracy.

## Discussion

In both our model and experiments, the peak calcium concentration evoked by somatically-generated action potentials decays with distance from the soma, suggesting that calcium signals could hold distance information for the synapse. To determine whether this was true for synaptically-induced BAPs, when the dendritic tree has a different level of excitability, we employed a computational model. The model predicts that for synaptically-driven BAPs, peak calcium concentration is the best correlate of synapse distance from the soma, both for synchronous and asynchronous synapse stimulation. This finding is robust for different dendritic morphologies. Furthermore, the functional measure of attenuation of the EPSP signal is also predicted well by peak calcium concentration. Importantly, homeostatic scaling of synaptic strength based on one target level of peak calcium for all synapses resulted in proximal synapses having lower strengths than distal synapses. Thus, peak calcium could be used for distant-dependent scaling of synaptic strengths and the establishment of a synaptic democracy.

The computational model used in this paper has multiple advantages over previous models and experiments exploring AP backpropagation. Firstly, due to technical limitations in experiments, backpropagation is always studied by direct stimulation of the soma to generate APs. In this situation, dendritic excitability is at resting state before the BAP arrives. In reality, dendrites are first locally activated by synaptic inputs, which in turn elicit an AP that backpropagates into the dendrites [6]. Synaptic activation has significant effects on local dendritic ion channels, modifying conductance states or even inactivating channels [13], [46] (Fig. S1, see also discussion below). Our model is synaptically-driven, with synapses dispersed across different branches, and takes into account the excitability due to local dendritic inputs. The initial synaptic stimulation changes membrane potentials in the spine via voltage-gated channels and induces calcium influx. The subsequent BAP causes an additional influx of calcium, mediated by R-type channels and NMDA channels that are unblocked by the initial spine activation (Fig. 2). All these effects do not occur with somatic stimulation alone (Fig. S1), which suggests that the results from BAP experiments previously described in the literature might differ from what occurs during *in vivo* synaptic stimulation. Secondly, in our study, in contrast to previous studies [47], [48], measurements are made in the spines, which are modelled as separate compartments with distinct channel composition from the dendrites. This is important biologically because the spine neck compartmentalises many biochemical signalling processes within the spine head [10], [19] and the spine head is likely to have a more direct influence on the local synaptic response than the dendritic shaft. Thirdly, in the model both calcium and voltage signals can be recorded simultaneously from multiple spines distributed across apical and oblique regions of the dendritic tree. Experimentally, this is currently not possible because it requires multi-photon microscopy that is fast enough to switch to and measure multiple spines within a millisecond time window. In addition, currently available voltage sensitive dyes have toxicity issues with low signal-to-noise ratio, and direct electrophysiological measurements in the spines are not possible. Lastly, in a model it is possible to incorporate adaptive changes in synaptic strength to investigate the development from a neuron with equal synapse strengths towards a neuron with scaled synapses. In experimental conditions this progression in time cannot be followed within the same cell.

Our model incorporates the current information available in the literature on ion channel densities and kinetics. This information comes from dendritic patch clamp recordings and electronmicroscopy immuno-labelings [15]. However, the original model of Poirazi et al. (2003) was optimised for a single morphology. To ensure that our results are not dependent on a unique morphology, we repeated the simulations for multiple morphologies (Fig. 3). The same main result was found for all morphologies tested, suggesting that our conclusions for peak calcium can be generalised to the general CA1 pyramidal cell population. In addition, the results were robust to different values of the AMPA conductance (Fig. S2). Note that our model had only excitatory synaptic inputs and did not include other types of input, such as GABAergic inhibition [49], [50]. The effects of these inputs need to be addressed in another study.

Synaptic democracy could be beneficial for memory encoding in CA1 pyramidal neurons. With a synaptic democracy in place, an effective synaptic strength can be encoded equally well at a distal synapse as at a proximal synapse, so that a neuron will have many equivalent synapses at its disposal. Another type of large pyramidal cell, the layer 5 neocortical pyramidal cell, does not have synaptic democracy [51]. This may be understood from a functional perspective: layer 5 pyramidal cells receive axonal inputs from different layers, each impinging at different but highly localised areas of the dendrite. These inputs convey different types of information and need to be processed differently, either via classical EPSP summation at the soma or via NMDA or calcium spikes in the dendrites [49], [52]. In contrast, CA1 pyramidal neurons receive axonal inputs from a single layer, CA3, and the inputs are widely dispersed over the dendrite [1]. If all these inputs need to be treated equally, CA1 neurons would benefit from a synaptic democracy.

Distance-dependent synaptic scaling was first shown in slice preparations using combined dendritic and somatic patch recording [3]. London and Segev [53] challenged whether the synaptic scaling observed *in vitro* could give rise to a true synaptic democracy *in vivo*, pointing out that slice preparations have artificially low levels of synaptic background activity. Their computational model showed that the scaling seen *in vivo* [3] would be insufficient to counteract the attenuation when *in vivo* like background activity is present. However, in contrast with our model, their model did not contain active channels, which, crucially, affect EPSP attenuation, and they used only subthreshold synaptic stimulation. In our model, background activity is present in the form of suprathreshold synaptic stimulation that triggers a BAP.

The conclusions from our model simulations should be regarded as a hypothesis, which could be tested experimentally once the field has developed a suitable technique to stimulate many synapses simultaneously to elicit an action potential. The most promising technique for doing this is glutamate uncaging combined with a piezo-controlled laser system that can quickly jump from one spine to the next to stimulate and measure hundreds of spines simultaneously [54]. We tested whether a few activated spines together with a somatically-induced action potential would be able to mimic a synaptically-induced BAP in an experimental setting. Compared with a synaptically-induced BAP, both a somatically-induced BAP and a combination of a somatically-induced BAP and synaptic stimulation produced a different voltage pattern across the dendrites (Fig. S1). Peak calcium is lower and the spread of membrane depolarisation into the oblique dendrites is different. In contrast, synaptic stimulation combined with a somatically-induced BAP would be a valid way to mimic the calcium signals in spines after a synaptically-induced BAP (Fig. S1D, I). The calcium signal in spines induced by a somatically-evoked BAP is only a fraction of what is induced by a synaptically-evoked BAP, with some distal synapses even lacking calcium influx altogether (Fig. S1M). However, note that a somatically-evoked BAP alone combined with the stimulation of a few synapses will lead to a great variability in spine calcium concentrations, depending upon the precise timing of the BAP relative to synapse activation across the dendritic tree.

In general, the timing and amplitude of signals are employed in a wide range of biological processes, such as protein synthesis and gene regulation [31], [55]. For neurons, membrane voltage and calcium concentration are important signals, which can open ion channels, evoke action potentials, trigger signalling cascades and activate protein synthesis [32], [56]. We therefore investigated which aspects of the calcium and voltage signals, namely their peak, integral and timing, could be used to establish a distance-dependent synaptic scaling (synaptic democracy). Surprisingly, we found that the peak voltage of the AP as it backpropagated into the dendritic tree was not a strong predictor of synapse location. The main reason for this low predictability is the differential propagation along the apical shaft and secondary/tertiary dendrites. In the apical shaft, BAP amplitude decreases with distance, in agreement with our experimental data and that of others [7], [42], [47], but increases when entering the oblique dendrites [57].

The time delay between synapse activation and BAP arrival, important for spike timing-dependent plasticity (STDP), has been hypothesised to be a signal for creating a synaptic democracy [58]. We observed that the delay-to-peak calcium is a predictor for synapse location only in subthreshold conditions (Fig. 4P) and is weakly correlated with distance in suprathreshold conditions. In addition, using delay-to-peak calcium or delay-to-peak voltage as a distance indicator would be problematic because with both these features the correlation with distance is in opposite directions for sub- and suprathreshold conditions (Fig. 2L, Fig. 4P, Table 1). This implies that a scaling process based on time-delay features would receive conflicting information with alternate subthreshold and suprathreshold inputs.

**Table 1:** Summary of *R* ^2^ values and directions of correlations for different conditions of synaptic stimulation.

	Calcium (*R* ^2^ value)			Voltage (*R* ^2^ value)		
	peak	integral	delay	peak	integral	delay
**BAP**	−0.64	−0.56	+0.33	−0.24	−0.47	+0.37
**asynchronous BAP**	−0.65	−0.60	+0.32	−0.23	−0.50	+0.53
**scaled synapse**	−0.48	−0.24	−0.20	−0.24	−0.36	−0.04
**subthreshold**	+0.03	−0.25	−0.87	+0.12	−0.35	−0.33

The investigated synaptic stimulations are: BAP, BAP with asynchronous jitter, BAP with scaled synapses and subthreshold stimulation. The symbols +/− in the table indicate the direction of the correlation between the measured signals (calcium and voltage) and the features (peak response, integral and delay-to-peak) tested for distance-dependence from the soma and measured in the spines.

It was previously proposed that a reverse STDP rule is required to set up synaptic democracy, followed by a switch to classical STDP rules in the adult CA1 pyramidal cell [58]. However, many studies show that the hippocampus uses classical STDP rules at CA3-CA1 synapses in both juvenile and adult stages [59]–[61]. Thus, no experimental evidence exists to-date for a developmental switch. Moreover, reverse STDP rules only at juvenile stages would make it difficult to accommodate new spines, which are regularly created in the adult neuron [62]. These new spines would not be scaled and would therefore behave differently from the other spines. Another modelling study showed that a distance-dependent STDP rule could also result in synaptic democracy [63]. However this mechanism would still require an internal signalling of distance to the spines to set the distance dependence learning rules. Our study, in contrast, has the same learning rule for all synapses.

For non-scaled synapses, peak calcium in spines showed the strongest correlation with distance (Figs. 2J, 3, 4F), with higher calcium concentrations in proximal synapses than in distal synapses, suggesting that this feature could be used for distance-dependent synaptic scaling. The high correlation of peak calcium with distance can be explained by the integrative properties of calcium, reflecting the integral of voltage rather than peak voltage (Fig. 5). Peak calcium was also strongly correlated with EPSP attenuation. However, when the neuron received only subthreshold activation (i.e. no BAP was generated), peak calcium was much lower and did not show distance dependence (Fig. 4N, Table 1). Importantly, using one target calcium level for all synapses and a homoeostatic scaling of AMPA conductances based on peak calcium resulted in a neuron with a self-organising form of synaptic democracy.

Homeostatic scaling of synapses based on peak calcium is an attractive and biologically plausible mechanism for creating a synaptic democracy. Firstly, calcium has been shown to regulate protein transcription, protein modulation and protein insertion [55]. Recently, two studies have shown that AMPA receptor expression at the synapse is homeostatically regulated by calcium [31], [32]. Reduced levels of postsynaptic calcium stimulate the production of retinoic acid, which in turn increase AMPA conductance [32]. Conversely, increased levels of activity in individual synapses of hippocampal cultures resulted in decreased AMPA receptor expression in a NMDA-dependent manner [31]. Secondly, homeostatic scaling takes place on a time scale of days, allowing synaptic changes governed by mechanisms such as STDP to occur on a shorter time scale. Thirdly, the system is dynamic and the same scaling rule can be used for all synapses independently of distance, so that newly-created synapses can scale themselves within an existing synaptic democracy.

In summary, our synaptically-driven model suggests that peak calcium levels in the spines are a strong predictor of the distance of a synapse from the soma and the level of attenuation its EPSP undergoes. It is robust to varying levels of activity, different dendritic morphologies and applies to both larger apical dendrites and smaller distal and oblique dendrites. Our results show that a form of homeostatic synaptic self-regulation, in which the synapse can utilise the BAP-induced peak calcium to adjust its strength, results in a synaptic democracy, where all synapses are equally heard at the soma.

## Supporting Information

Figure S1 **Comparison of different BAP induction paradigms.** **A–E,** Synaptically-induced back propagation. **F–J,** Somatic action potential induced by somatic current injection 13 ms after a stimulation of a small number of synapses. The EPSP-AP delay was 16.1+/−1.7 ms compared to 16.6+/−0.7 ms in Fig. 2. **K–M,** Somatically-induced action potential. **A, F, K,** Illustration of the different BAP induction paradigms. Stars represent synaptic stimulation. Arrows indicate propagation to and from the soma. **B, G, L,** Correlations of distance to soma plotted against peak voltage measured at the spine. **C, H,** Correlations of distance to soma plotted against delay-to-peak voltage (defined as the time between the stimulation of the synapse and the peak of membrane potential). Note that this is not defined for synapses for the somatically-induced BAP as in K. **D, I, M, **Correlations of distance to soma plotted against peak calcium concentration. **E, J,** Correlations of distance to soma plotted against delay-to-peak calcium values.
(EPS)

Figure S2 **Correlations of features with distance are robust to increased synaptic AMPA conductance.** Synaptic AMPA strength at all spines was doubled from 200 pS to 400 pS. **A–F**, Peak, integral and delay-to-peak voltage and calcium signals correlations against path distance. Coloured circles indicate selected spines (see Fig. 2). *R* ^2^ values indicated for each fit.
(EPS)

Figure S3 **Synaptically-induced BAP without R-type channels.** **A**, CA1 model neuron. Circles indicate spine head locations of example measurements highlighted in B–L. **B–F**, Measurements from the spine heads indicated by the corresponding colours in panel A, indicating membrane potential (**B**), calcium concentration (**C**), NMDA-mediated currents (**D**), AMPA-mediated currents (**E**) and R-type currents (**F**). The right-hand plots show the same data, but magnified around the time of the arrival of the BAP at the spines **G–L**, Peak, integral and delay-to-peak voltage and calcium measurements are plotted as correlates for path distance. Black circles indicate apical shaft spines. *R^2^* values are shown.
(EPS)

Figure S4 **Individual synapse scaling in a homeostatic manner based on peak calcium levels.** **A**, Example traces of individual synapse strength during the first 200 runs of the timelapse homeostatic simulation (same simulation as in Fig. 8). Synapses are colour-coded according to soma-synapse distance. **B**, Examples of peak calcium levels for individual synapses during the time-lapse homeostatic simulation. On each run, a new set of synapses was stimulated. Synapses are colour-coded as in A, with individual points showing the fluctuations in peak calcium concentration around the target level following sub- and suprathreshold responses.
(EPS)
